# Flower strip networks offer promising long term effects on pollinator species richness in intensively cultivated agricultural areas

**DOI:** 10.1186/s12898-018-0210-z

**Published:** 2018-12-04

**Authors:** Constanze Buhk, Rainer Oppermann, Arno Schanowski, Richard Bleil, Julian Lüdemann, Christian Maus

**Affiliations:** 1 Institute of Agroecology and Biodiversity (IFAB), Böcklinstr. 27, 68163 Mannheim, Germany; 2 Institute for Environmental Sciences, University Koblenz-Landau, 76829 Landau, Germany; 3 Institut für Landschaftsökologie und Naturschutz (ILN), Sandbachstr. 2, 77815 Bühl, Germany; 4 Bayer AG, Bee Care Centre, 40789 Monheim, Germany

**Keywords:** Agri-environmental schemes, Bees, Butterflies, CAP reform, Floral resources, Flower-strips, Fragmentation, Long-term field experiment, Specialist species, Pollinators

## Abstract

**Background:**

Intensively cultivated agricultural landscapes often suffer from substantial pollinator losses, which may be leading to decreasing pollination services for crops and wild flowering plants. Conservation measures that are easy to implement and accepted by farmers are needed to halt a further loss of pollinators in large areas under intensive agricultural management. Here we report the results of a replicated long-term study involving networks of mostly perennial flower strips covering 10% of a conventionally managed agricultural landscape in southwestern Germany.

**Results:**

We demonstrate the considerable success of these measures for wild bee and butterfly species richness over an observation period of 5 years. Overall species richness of bees and butterflies but also the numbers of specialist bee species clearly increased in the ecological enhancement areas as compared to the control areas without ecological enhancement measures. A three to five-fold increase in species richness was found after more than 2 years of enhancement of the areas with flower strips. Oligolectic bee species increased significantly only after the third year.

**Conclusions:**

In our long-term field experiment we used a large variety of seed mixtures and temporal variation in seeding time, ensured continuity of the flower-strips by using perennial seed mixtures and distributed the measures over c. 10% of the landscape. This led to an increase in pollinator abundance, suggesting that these measures may be instrumental for the successful support of pollinators. These measures may ensure the availability of a network of diverse habitats and foraging resources for pollinators throughout the year, as well as nesting sites for many species. The measures are applied in-field and are suitable for application in areas under intensive agriculture. We propose that flower strip networks should be implemented much more in the upcoming CAP (common agricultural policy) reform in the European Union and promoted more by advisory services for farmers.

**Electronic supplementary material:**

The online version of this article (10.1186/s12898-018-0210-z) contains supplementary material, which is available to authorized users.

## Background

Sustainable development of agricultural landscapes is essential for global food security [1, 2] and for the provision of a safe operating space for humanity [3]. Sustainable agriculture strongly depends on ecosystem functioning, which is linked to landscape diversity including biological, crop and management diversity [4, 5]. About half of Europe’s territory consists of agricultural land [6]. Farming practices in Europe have been intensified in the past decades [6] a process which continues in many regions [7]. Agricultural intensification is linked to high yields and low labour inputs, however it is regarded as one of the main factors implicated in the widely reported decline of biodiversity and insect abundance in agricultural landscapes [8–10] and of ecosystem functioning [4, 11]. Ecosystem functions like water and nutrient storage capacity, carbon sequestration, pollination services and pest control or protection against extreme weather events [5] tend to be linked to the variety of species traits and the multifunctionality of high biodiversity [12–14].

The rapid loss of biodiversity and ecosystem functioning in agricultural areas [15] calls for quick and effective countermeasures to prevent the regional loss of species pools in the intensified agricultural landscape [16]. Many such countermeasures have been evaluated for their effects on biodiversity loss [17–20]. Several studies have come to the conclusion that agri-environmental programs designed to halt biodiversity loss in the agricultural landscape in Europe are not always effective though they may be expensive and labour intensive [10, 21–23]. This is one of the reasons why the programs are implemented only to a small extent—especially in highly productive areas [22]. Measures can be ineffective if they are too far apart (i.e. fragmented), implemented on too small an area, or not adapted to the landscape matrix, i.e. if the species they are targeted at are not present [21]. Therefore, new and more efficient approaches need to be developed to give incentives and precise advice to farmers, on how diversity loss may be halted efficiently or how diversity could be restored. For this, it will be helpful to find realistic, adaptable and quantifiable approaches for farmland management methods, which are (a) easily applicable, (b) highly effective and (c) allow quick acceptance and action of farmers in intensively used landscapes. If such measures would be widely applied throughout the intensively used agricultural areas, this could counteract a loss of ecosystem functions like water and nutrient retention capacity or pollination services as well as regional species losses. These measures alone will not be sufficient to preserve biodiversity in the agricultural landscape as a whole, but they may be an important element in making farming more sustainable.

Here, we present and evaluate such an easily applicable measure to increase biodiversity in intensively used agricultural landscapes. Flower strips are widely used throughout Europe but come in many different forms and their effects on biodiversity vary strongly [24]. We demonstrate the effects of perennial flower strip networks for pollinator diversity. Pollinators (wild bees and butterflies) are used as target species to evaluate the efficacy of the presented measures as these groups are especially functionally relevant and include many species sensitive to intensification [11, 25]. In addition, wild bees are known to react sensitively to isolation of foraging habitats [26, 27] and the amount of flowering resources directly [28, 29]. The key characteristics of the tested enhancement measures are:

(i) the sowing of multiple flower strips in the landscape with a total extent of 10% in 50 ha study areas to avoid effects of fragmentation of food and nesting resources.
(ii) high plant species richness and complementarity of different seed mixtures to provide food also for specialist pollinators (bees, butterflies and butterfly larvae).
(iii) the sowing early and late in the year of annual and perennial species mixtures to ensure the provision of a high variation of food over the year as well as hiding and nesting sites. Most strips remained in place over several years.

Our practical experiment and evaluation differs from most other studies evaluating impacts of environmental schemes like flower strips, which often either lack repetition, are only observed for relatively short time periods, lack reference data before the enhancement measures took place and refer to single strips or even strip sections [22, 30]. Only a few studies include data from multiple years [10]. Further, often just one single group of species is evaluated [31]. Here, we present a replicated study surveying two large species groups (quantitative data on wild bees and presence/absence data on butterflies) at five times per year for a period of 5 years. We also surveyed comparable control areas in order to evaluate the effects of the in-field conservation measures applied, and collected reference data from the year before the enhancement measures were generated. The data refers to the effects of a network of flower strips at the landscape scale (50 ha) and is not restricted to effects of individual flower strips, which has rarely been done so far (but see [32]). We hypothesise that

- The implementation of a flower strip network increases local abundance of bees (all and oligolectic only) and diversity of bees (all and oligolectic only) and butterflies.
- The positive effects of the implementation of a flower strip network on local abundance and diversity of bees and butterflies increase with the time since establishment.

## Methods

### Study area

**Fig. 1:**
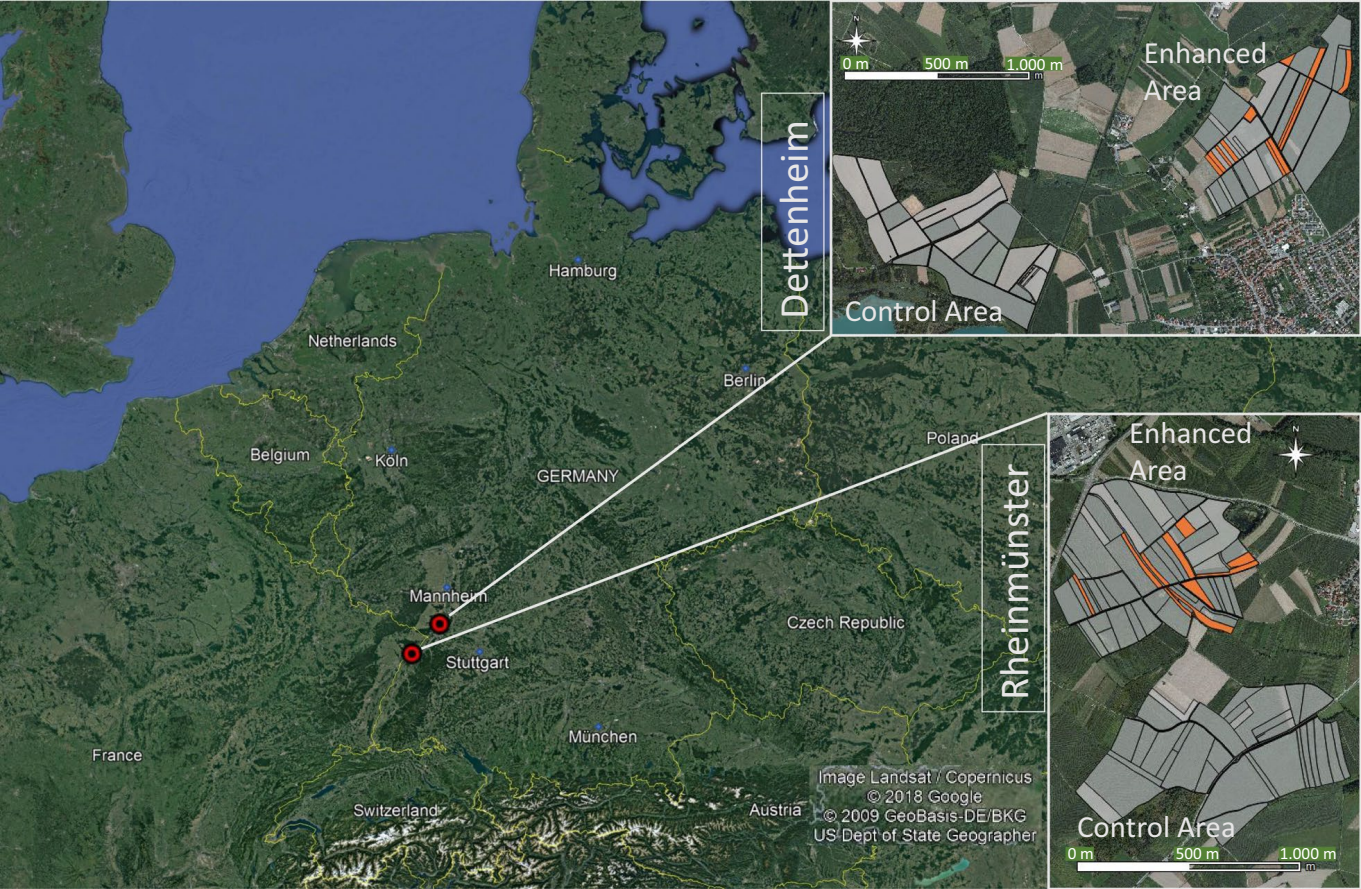
Location of the study regions near Dettenheim and Rheinmünster in Southern Germany in Central Europe. The study areas are marked in red. Larger cities are marked in blue. Flower strips located in the enhancement areas are marked in orange

The project was conducted at two sites in the Upper Rhine Valley in the German Federal State of Baden-Württemberg; the Bolzhof farm in Dettenheim and the Birkenhof farm in Rheinmünster (Fig. 1). Both land-owners kindly agreed to cooperate. Two 50-hectare study areas were established at each site; one to test the enhancement measures and the other one as a control area. The project sites are located in intensively farmed agricultural landscapes comprising around 95 percent arable land (mainly maize and winter cereal cultivation) and very little grassland. Detailed maps of land-use in 2015 are available as Additional file 1. Though the fields are intensively used, the landscape is only moderately simplified as is typical for Southern Germany, field sizes are small to intermediate (about 1 to 8 ha), compared to North-Eastern Germany or Eastern Europe where field sizes are much larger, leading to a reasonably high number of edge structures and paths. Further, semi-natural structures like hedgerows and taller vegetation structures along ditches occur. Therefore, we consider our region as intensively managed, but with intermediate simplification.

### Ecological enhancement measures

In the first year of the study, 2010, a baseline survey was conducted in the control areas as well as in the designated enhancement areas; in this year, no ecological enhancement measures were carried out. From 2011 onwards, mixtures of cultivated and wild flower seeds were sown on five hectares of the enhancement areas (10%), while no enhancement measures were carried out in the control areas (Fig. 1; Additional files 1, 2 and 3).

#### Description of enhancement measures 2011–2015

In the first year of the enhancement measures, four different commercial, annual flower mixtures were sown in late spring (early May). Seeding history for all strips is shown in Additional file 2. No measures were implemented on the control study areas. From the second year onwards, in addition to the spring sowing, winter-hardy seed mixtures were sown in autumn including annual and perennial species (September/October). From autumn 2012 onwards, mixtures of predominantly perennial flowers were sown on plots alongside the annual and winter-hardy mixes. Areas sown in autumn and perennial strips provide food for insects in early spring March/April. The decision to re-seed a strip or not was a decided by the farmers and the project coordinator depending on the degree of development of various flowers or problematic weeds. To keep the system flexible, no strict rules were applied concerning flower strip mulching and/or reseeding times. Typically, strips were not resown if they remained diverse but if they were invaded by weeds then at least parts of a strip might be resown.

An important consideration for the project has been an appropriate distribution of flower patches in space and time. This ensures a network of flower patches and strips in the landscape, which reduces the travelling distance for pollinators between the patches to a minimum and provides food sources and hiding places throughout the year (see scheme in Fig. 1 and maps in Additional file 1).

### Sampling

In 2010, the designated study areas were recorded in a baseline survey to determine the current land-use and the populations of wild bees and butterflies before enhancement measured were conducted. In the years following the implementation of the ecological enhancement measures in 2011, similar surveys were carried out every year.

Sampling of bees and butterflies was conducted between mid of April until mid of August by observation and by collection of specimens with sweep nets. In each year, after a first overview survey of the 50 ha areas, four (in control areas) and five (in enhancement areas) of the most diverse structures were chosen for the annual sampling. The most diverse structures were flower strips in the enhancement areas and field edges and grassy paths in the control areas. To account for daytime variations, sampling was done twice a day. Sites mapped during less favourable times in the early morning were sampled during the most favourable times in the early afternoon and vice versa, to achieve a balance of sampling quality across the day. Per year and site, five surveys were conducted at different times of the season. Control and enhancement areas were sampled within a few days depending on weather conditions and man-power available (Additional files 4 and 5).

Wild bees were studied by walking through the chosen sampling structures for half an hour twice a day scanning diverse floral resources for bees. Some species could be determined without capturing them and in this case their abundance, sex and fodder plants were recorded directly. Others were captured and stored in small pots. After 30 min recording time some bees could be determined using a magnifying lens and could be released. Remaining individuals were killed in ethyl-acetate, transported to the lab, fixed and identified.

Butterflies were only recorded qualitatively as presence/absence data between 9 am and 4 pm at a maximum distance of 5 m along a line of about 250 m (transect sampling) within the selected structures. Observations pointing to sedentariness like oviposition, the presence of caterpillars and egg laying activities were recorded. The original plan, to sample semi-quantitatively along the transect, had to be abandoned as it was impossible to walk along a line and cover 5 m of width due to the density of the vegetation within some of the flower-strips. Most butterflies were determined by sight. If this was not possible, they were captured with a swap net for later identification.

Species identification followed Amiet [33], Amiet et al. [34–38], Dathe et al. [39], Scheuchl [40, 41], Scheuchl and Schwenninger [42], Schmid-Egger and Scheuchl [43] for bees and Settele et al. [44] for butterflies. The categorization of the bees into oligolectic versus polylectic species followed Westrich [45].

### Statistical analyses

Change through time in species numbers and abundances were plotted in between 2010 (the starting point before the enhancement measures were applied) and 2015 (the fifth year of enhancement measures). The data of the two regions were analysed separately. The data collected in different surveys (five sampling dates) was pooled over the year to achieve year-specific numbers.

As there were year-specific fluctuations in insect abundances depending on the weather conditions, the control area values were subtracted from the enhancement area values to compare the overall effect of the enhancement over the study period. We compared the differences between 2010 and 2015 to see the weather independent effect of the enhancement in 2015, relative to the reference period before the enhancement in 2010. To do so, the data were again pooled over the 5 sampling events per strip and year. The mean number of species or individuals in the control area was calculated and then subtracted from each of the values collected in the enhancement areas for each parameter and for the years 2010 and 2015 separately. This difference measure, Delta (D), was then compared, using Mann–Whitney-U-tests, between the original situation in 2010 and the situation in the year 2015. Non-parametric U-tests were used, as most datasets were not normally distributed and/or variances were not homogenous. Dependent variables were the number of species and abundance of bees, the number of oligolectic species and abundance of bees, the Chao estimate of bee species diversity (Chao1) and butterfly species numbers and diversity (Chao2). The Chao Diversity estimates were calculated to compare the diversity of the areas incorporating the different sampling effort in the 2015 enhancement areas in contrast to the controls. Chao1 was used for abundance data and Chao2 for presence/absence data. The Chao Diversity estimators were calculated using EstimateS 9.1. [46]. Graphical presentation and U-tests were done using IBM SPSS Statistics Version 25 [47].

## Results

**Fig. 2:**
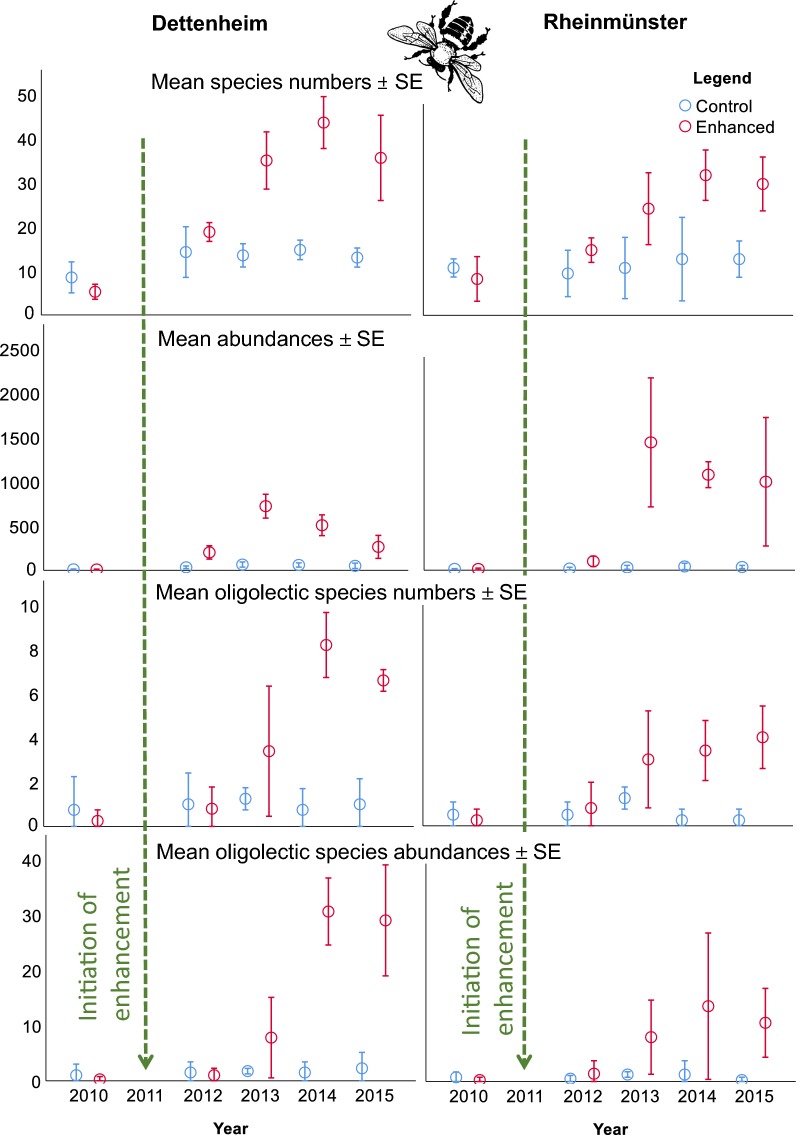
Change through time of bee species richness and abundances between 2010 (before the enhancement measures) and 2015. Upper graphs refer to all species, lower graphs refer to oligolectic species, only. The green line marks the year 2011 when the enhancement measures were initiated; blue symbols: control area; red symbols: enhancement area. The clipart is freely available via https://openclipart.org

Bee species richness and abundance including oligolectic species clearly increased in the enhancement areas during the 4 years of observation while the enhancement measures were applied. This is indicated by the increasing means and standard errors, which do not overlap after the second or third year depending on the location and parameter (Fig. 2). No statistical tests were carried out due to the low level of replication. There was no further systematic increase after 4 years of observation. Especially from the second year, 2013, onwards, bee abundance was high but fluctuated strongly.

**Fig. 3:**
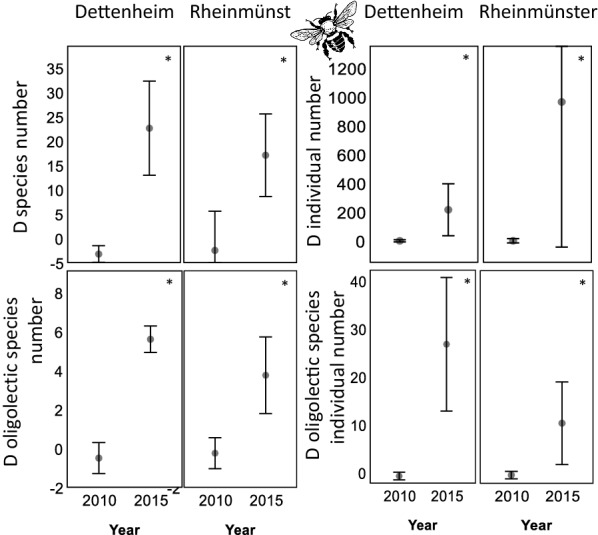
Differences D of species numbers and abundance of bees between enhancement area and control area. Comparison between the enhancement areas (EA) and the control areas (CA) in 2010 (before the enhancement measures started) and in the year 2015 (in the fifth year of enhancement measures). The values present the mean difference D between the enhancement area and the control area ± standard error, therefore slightly negative values or overlapping standard errors are possible. Significant differences between the years 2010 and 2015 are marked with * if p < 0.05; ** if p < 0.01 and *** if p < 0.001. The clipart is freely available via https://openclipart.org

**Fig. 4:**
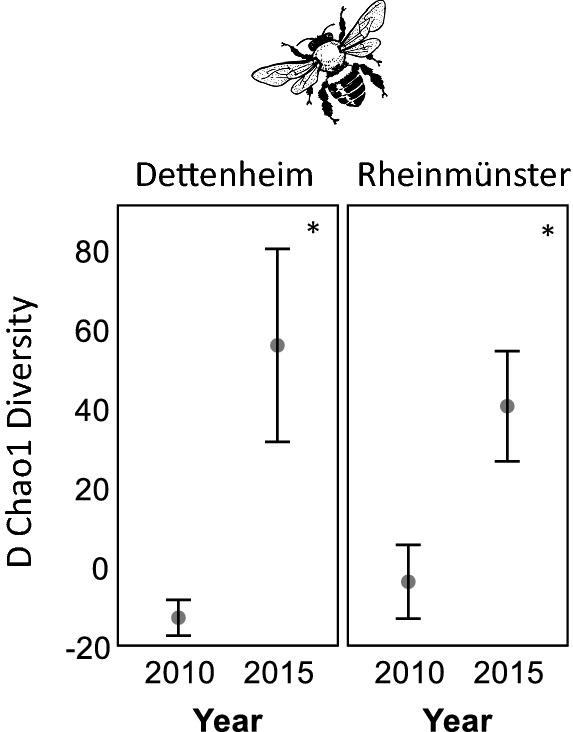
Difference D of the Chao 1 Diversity of bee species. Comparison between the enhancement areas (EA) and the control areas (CA) in 2010 (before the enhancement measures started) and in the year 2015 (in the fifth year of enhancement measures). The values present the mean difference D between the enhancement area and the control area ± standard error. Significant differences between the years 2010 and 2015 are marked with * if p < 0.05; ** if p < 0.01 and *** if p < 0.001. The clipart is freely available via https://openclipart.org

The number of bee species and their abundance in 2010, before the enhancement measures, were very similar between the enhancement areas and the control areas as can be seen in values close to zero in Fig. 3. In 2010, before the enhancement measures, there was even a trend towards slightly higher bee species numbers in the control areas as the negative differences D between species in EA and CA in Fig. 3 indicate: The initial richness was on average lower in the area that was enhanced in 2011 than the control area in 2010. Species numbers increased significantly (according to Mann–Whitney U-tests) by more than 20 bee species in Dettenheim (p = 0.014) and by about 15 bee species in Rheinmünster (p = 0.014), in the enhancement areas in 2015 as compared to 2010 (Fig. 3). This corresponds to a three to five-fold increase. Bee abundances (Dettenheim: p = 0.014; Rheinmünster: p = 0.014), oligolectic species numbers (Dettenheim: p = 0.011; Rheinmünster: p = 0.013) and abundances (Dettenheim: p = 0.013; Rheinmünster: p = 0.012) increased significantly over the study period in both locations (Fig. 3). Chao1 Diversity estimates increased significantly in both areas (Dettenheim: p = 0.014; Rheinmünster: p = 0.014; Fig. 4).

Highly endangered species at a national scale (red list Germany) were only found in the enhancement areas. A category 2 (“critically endangered”) species *Lasioglossum pauperatum* was found in both locations and *Andrena limata* was also found in Dettenheim. A category “R” (“very rare”) species was found in Rheinmünster in 2015 (*Anthidium septemspinosum*). There were 52 endangered or locally vulnerable species (according to the red list of bees in Baden Württemberg) in Dettenheim and 37 in Rheinmünster with 32 and 23 species respectively which were restricted to the enhancement areas. Most oligolectic bee species, mainly female individuals, could be observed collecting their specific source of pollen in the flower strips. These were mainly species that are specialized to collect pollen on Fabaceae (like *Andrena lathyri, Eucera nigrescens, Megachile ericetorum*), Apiaceae (like *Andrena fulvicornis, Andrena nitidiuscula, Andrena rosae)*, Asteraceae (like *Colletes similis, Dasypoda hirtipes, Heriades crenulatus*) and Brassicaceae (like *Andrena agilissima, Andrena distinguenda, Osmia brevicornis*) and a single species collecting solely on *Ranunculus* (*Chelostoma florisomne*) or on *Reseda* (*Hylaeus signatus*). Purely nectar-consuming individuals of oligolectic species were also observed.

**Fig. 5:**
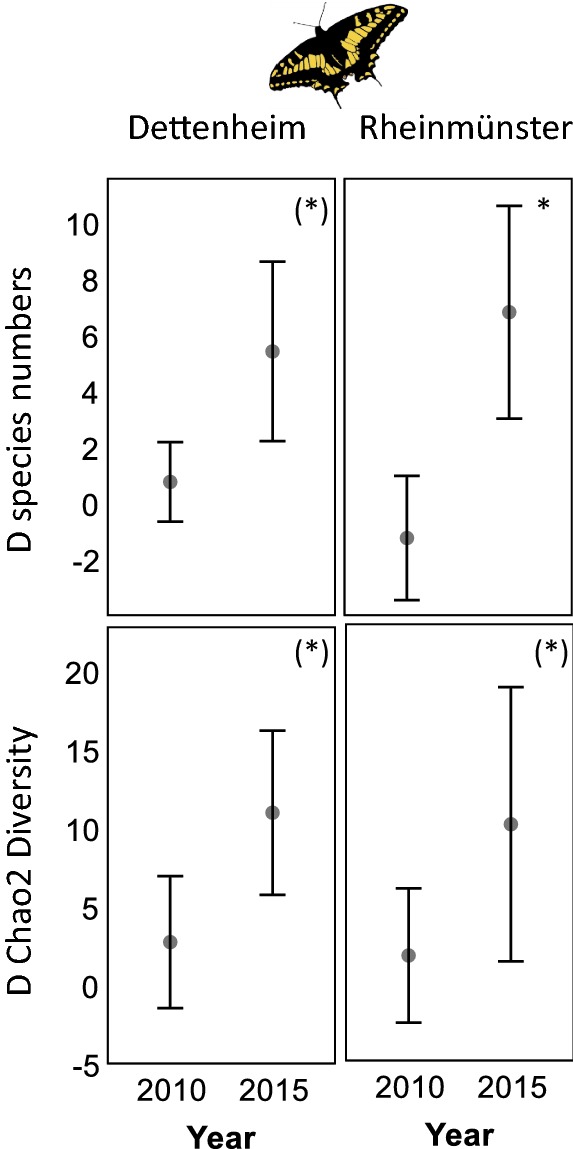
Difference of species numbers and Chao2 Diversity of butterflies. Comparison between the enhancement areas (EA) and the control areas (CA) in 2010 (before the enhancement measures) and in the year 2015. The values present the mean difference D between the enhancement area and the control area ± standard error. Significant differences between the years 2010 and 2015 are marked with * if p < 0.05; ** if p < 0.01, *** if p < 0.001 and (*) if p < 0.01. The clipart is freely available via https://openclipart.org

In 2010, before the implementation of the enhancement measures, the mean numbers of butterfly species recorded per transect (mean (± standard deviation)) were 3.6 (± 1.4) species in Dettenheim and 5.4 (± 1.9) species in Rheinmünster. In total, 6/7 species were found in the 2010 control/enhancement area in Dettenheim and 10/10 species in Rheinmünster before the enhancement. The highest total butterfly species numbers in the enhancement areas were found in 2013, with 21 species in Dettenheim and 18 species in Rheinmünster. In 2014 and 2015, however, the species richness in the enhancement areas again decreased slightly. Comparing the effect over the whole study period, butterfly species numbers were significantly (according to Mann–Whitney U-tests) higher in 2015 with on average 7 species more in Rheinmünster (p = 0.014) in the enhancement areas per sample transect compared to the control areas (Fig. 5). In Dettenheim, a marginally significant increase (p = 0.085), of on average 5 species, was found between 2010 and 2015. The variation between the individual samples in 2015 was high. Overall species numbers in 2015 in the enhancement areas reached 16 species in Dettenheim and 23 species in Rheinmünster, versus 6 species and 10 species respectively in the control areas. In both areas, a marginally significant increase in Chao2 diversity of butterfly species was found (Dettenheim: p = 0.086; Rheinmünster: 0086; Fig. 5).

Several species of butterflies were found to reproduce in the flower strips as egg-laying individuals and caterpillars. Endangered or locally vulnerable species (according to the red list of butterflies in Baden-Württemberg) found as caterpillars, eggs or search for site to lay eggs were *Carcharodus alceae, Colias hyale, Cupido argiades, Erynnis tages, Leptidea sinapis s.l., Lycaena dispar* and *Lycaena phlaeas*. Other prominent locally reproducing species were *Papilio machaon* or *Vanessa cardui*. Food plants of the caterpillars of several butterfly species found were not present in the flower strips.

## Discussion

**Fig. 6:**
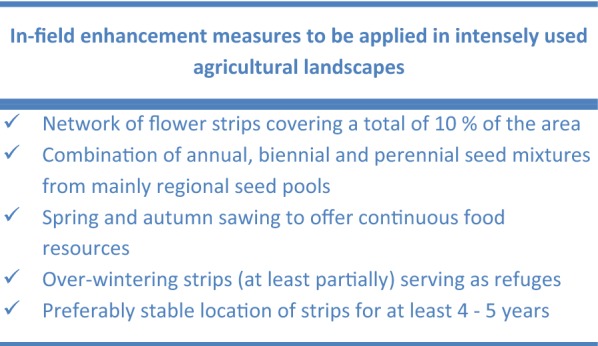
Summary of relevant aspects of flower strip networks that increase pollinator species richness. The described set of easily applicable in-field measures builds upon the experiences of existing theoretical and applied studies and was evaluated within this study

The flower strip networks, covering 10% of the 50 ha areas, had significant effects on the species richness and abundances of wild and oligolectic wild bees as well as the number of butterfly species. This may be the result of a number of characteristics of the studied flower strip networks that were considered during setup, as flower strips are not always as effective as in this case [48]. Here, several crucial measures to improve pollinator abundance and diversity were applied simultaneously (see Fig. 6), namely: that a relatively large area (10%) of the landscape was covered with flowering strips, which is known to favour pollinators through the elevated amount of pollen and nectar [28, 29]; that the distances between the strips were kept low enabling less mobile species to also reach the resources [18, 49]; and that a variety of seed mixtures and temporal management options were applied to increase resource continuity and variety, which has previously been shown to be important [50].

### Spatial arrangement of food and habitat for pollinators

Some species like the common bee species *Osmia bicornis* are very flexible regarding their diet and nesting sites [51], however, this flexibility is probably limited when there is a lack of suitable structures and when distances between food sources become too large. Small-sized pollinators are particularly strongly range-limited [49, 52]. Long distances between food sources might not directly affect the presence of the species, but could alter their abundance and reproductive success [53]. Carvell et al. [54] found higher reproduction of bumblebees when resource patches were larger (1 ha) allowing shorter flight distances to reach floral resources, and Coudrain et al. [51] showed that parasitism of *Osmia bicornis* increased with longer flight distances, as the nests remained unguarded for a longer period of time. More specialised species are especially affected when the distances between their specific food sources are high, when their quality is low, and when their temporal continuity is short [11, 25] According to our data, the network of flowering strips created in this study is able to enhance also specialist species richness and abundance, which is not the case in other flower-strip studies [55]. The plant species selections in the seed mixtures contained many species and also provided food for specialist bees [56, 57]. Specialised species play an important role in determining the functional diversity of a pollinator community and tend to suffer more from land-use intensification, than polyphagous species [18]. Pollinator species richness and the number of species in flower mixtures are known to be positively correlated [55].

### Temporal continuity of food and habitat for pollinators

Besides the good connectivity between the strips and the species rich flower mixtures used, the diversity of habitats created by different strip longevities and seeding times might also have contributed to their success [58], as they lead to continuity and a large variety of foraging options [29, 59]. To provide a high variation in food, hiding and nesting sites over the year to support all life cycle stages, some flower strips were sown early and some late in the year. Some strips remained untouched over the winter after flowering had ceased to ensure the presence of undisturbed bare soil, dry inflorescences and stems as hiding places for all sorts of insects over the winter and consecutively for fast re-colonialization in the following year [60]. To support species that are active during different time periods over the year, floral resources must be high and diverse over the whole vegetation period. This is achieved by variability in sowing times and thus main flower development phases [52], as well as different types of overwintering flower strips. This also increases the likelihood of forage being available, independent of the specific weather and site conditions.

The development of the flowering strips was monitored regularly during the project. This was necessary to ensure the quality of the individual flowering strips and their species composition in the landscape, and thus to achieve spatial and temporal continuity of food availability also for specialized bee species. Seed mixture germination success and resulting vegetation composition can be very variable depending on site and weather conditions. Therefore, in this study, it was not defined beforehand which strip should be managed in which way, this was decided on the basis of observations over the year. However, perennial seed mixtures and overwintering flowering strips, or at least parts of the strips always made up a large proportion of the flower strip network (Additional file 2). Further, seed mixtures were continuously adapted to site conditions and various functional groups of flowers and wild forms of species were favoured over cultivated plant taxa such as *Helianthus annuus*. If a strip developed monodominant stands of e.g. *Phacelia tanacetifolia* or large patches of species known to be aggressive weeds (e.g. *Cirsium arvense)*, the strip was reseeded instead of being left over winter at least in parts of the strip. Such flexibility is an important requirement for the implementation of the measures to increase their effectiveness and ensure their acceptance at the same time. Our data show that rare and specialist bee species can clearly profit from this approach. While effects were small in the first year after the flower strips were created (2012), the consecutive years clearly increased and fluctuated at a high species richness and abundance level. This underlines the relevance of long term observation on the one hand but also of the effectiveness of the enhancement measure over time scales longer than 2 years. Specialist species profited especially from the longer duration of the measures and/or from the many overwintering strips. Sowing in autumn helped to increase early season flower abundance and suppressed the appearance of unwanted weeds in spring like the spring germinating species *Amaranthus retroflexus*, *Echinochloa crus*-*galli* or *Chenopodium* species.

Flower strips in Rheinmünster were especially dominated by a few bumblebee species like *Bombus terrestris* with 2300 individuals in a single sample in the enhancement area in Rheinmünster in 2015, which was nearly 50% of all individuals found. Together with the very common *Bombus lapidarius* (about 20%) and *Bombus pascuorum* (13%), these three species made up over 80% of all individuals found. These generalist bumblebees may have been attracted by very dense cover of red clover (*Trifolium pratense*) in 2015. More specialized species were lower than average in the same strips.

### Flower strips in the agricultural landscape

The ecological enhancement measures presented here are not thought to replace other well-known ecological enhancement measures that are applied either in field (like the use of fallow phases) or at the field borders like species flower-rich field margins or in some regions the plantings of hedgerows that serve as refuges [25]. Especially butterflies seemed to depend on semi-natural structures in the direct neighbourhood. The measures, flower strips and semi-natural structures, mutually enrich each other as permanent structures serve as refuges and sources for species while short term structures like various flower strips connect habitats and provide essential all year long food resources. Flower strip networks may therefore also contribute to counteracting the vicious cycle of limited floral resources reducing bee pollinator abundance and pollinator decline in turn reducing animal pollinated plant abundance [61]. Optimally, local seed pools should be used to seed the flower strips to strengthen regionally adapted forms of wild plant species and to strengthen local floral diversity and pollen resources [62]. Thus, an increase in pollinator species richness might secure the reproduction of pollinator dependent flowering plants in adjacent semi-natural areas that frequently suffer from fragmentation [19, 20].

Such field strip networks are designed to be effective within a conventionally managed agricultural landscape. However, the indiscriminate application of pesticide in the direct surrounding of flower strips could reduce the value of the measures due to pesticide drift (potentially about 30% of the in-field application rate within 1 m from the field [63]). The ingestion of contaminated pollen and nectar from some pesticides over longer time periods may lead to concentrations that are lethal or sub-lethal for bees and their larvae [61, 64–66]. However, the strong increase in species numbers and abundances in our study demonstrates that such effects were not relevant in the study areas indicating that in these cases, conventional management followed “good agricultural practice”, which avoids drift of toxic pesticides to the flower strips. We cannot exclude the possibility that agronomic inputs may have influenced the pollinator populations in this study but we can conclude that positive effects were seen despite any possible effects. Sensitivity of the farmers concerning the type of pesticides and the application method are therefore important prerequisites of successful flower strip networks. By avoiding the contamination of flower strips through insecticide drift, the farmer may benefit from higher natural pest control from the field strip to the adjacent crop [67, 68].

The ecological enhancement measures described here help to keep diversity and abundances high, provide habitat for several endangered species and may help to enhance certain ecosystem functions in intensively used agricultural areas [69]. For the protection of certain particularly sensitive specialist species, habitats like extensively used land-use systems, such as semi-natural grassland areas are probably irreplaceable [55]. The large increase of also specialized and endangered species in the flower strip networks is in contrast to the review of Scheper et al. [55], who stated that many flower strip approaches just lead to the success of the most common and non-specialized pollinator species. Here, the set of different measures, including temporal dynamics of measures and an adapted plant species composition, implemented in our study probably makes the difference. For butterflies, the increase of species richness was less obvious, which is in line with studies from agricultural areas in Switzerland [70] and from Finland [71]. In the latter study, regions of over 60% of agricultural area are clearly impoverished in butterfly richness and the enhancement of 10% of the area might therefore not be sufficient to attract more specialized species.

Implementing flower-strips at 10% of the agricultural area may sound a lot to many farmers. However, the area of 8% of the farm being used as perennial flower strips has already been demonstrated to lead to no net loss of monetary income or food quality as ecosystem services were increased [69]. In this case, it took about 4 years until the beneficial effects were large enough to compensate for the reduced area under production. This is in line with our observations that specialist species, which are known to be especially relevant to offer ecosystem services [4], needed 3 to 4 years to establish.

### Transferability

The two landscape sections chosen for the ecological enhancement measures clearly show impoverishment of the flora and fauna due to agricultural intensification, but they still contain semi-natural habitats and extensively used areas within a 500 m buffer around the study areas. This is important for the efficacy of the measures, as landscapes that contain no natural or semi-natural structures may have already lost their regional species pool. Reestablishment of a diverse pollinator community may therefore not be possible within the studied time span [21, 29]. Especially for butterflies, structures close to forest and hedgerows clearly increase species richness. Accordingly, in regions with large-scale, homogenized agricultural landscapes such as those found in parts of North-Eastern Germany, the measures described would have to be accompanied with parallel restoration activities in natural habitats and potentially with species reintroductions [72]. Our study is in line with Tscharntke et al. [73] who show that several small habitat fragments that are connected may be as effective as large fragments in conserving specialist species diversity, and that measures such as flower strips are most effective in landscapes of intermediate levels of simplification [74]. Transferability of the success of the enhancement measures might therefore be high. However, as we used this diverse set of different measures as a whole (summarised in Fig. 6) we cannot tell which aspect (e.g. total area, configuration of strips, duration of strips, seed mixtures, and combination of strips with other semi-natural habitats) was most important in causing the positive effects found. However, all aspects have been proven to be individually relevant in the cited literature. Our results therefore suggest that a combination of the aspects similar to the enhancement method tested here, might be effective in many other situations in similar landscapes and not just in the two case studies reported here.

## Conclusions

Dicks et al. [75] formulated a minimum goal to conserve some biodiversity and ecosystem functioning in agricultural landscapes. They claim that minimal action is needed to support pollinators on farmland but their measures only favour the most common generalist bee species. This might ensure pollination services for principal crop plants under stable environmental conditions [76]. In line with Burkle et al. [77], however, we follow the call for approaches to support a more diverse pollinator community to foster multifunctionality [4, 78] and to avoid dependency on a few phylogenetically strongly related generalist species that might be sensitive to negative effects of newly emerging diseases or extreme weather events (compare to the insurance hypothesis, [5, 79, 80]).

To increase the acceptability of ecological enhancement programs to farmers, the measures should be made attractive (including by a reduction of bureaucratic obstacles and sufficient incentives especially in highly productive areas) and designed to cover a sufficiently large extent of the landscape. Implementation is facilitated when the measures are individually scalable and flexible over time, allowing for spatial and temporal adaptations over time without much organizational effort. Accordingly, the methods described here can be easily applied by the farmers, which can make them quickly applicable even in large areas. In order to prevent the loss of insect biodiversity in general and pollinator species in particular in the agricultural landscape, the adaptation of respective ecological enhancement measures by a majority of the farmers would be advisable. Such measures could for instance be integrated into a broadly implemented agri-environmental policy e.g. within the European CAP. Most farmers, however, need support and specific advice to be reactive in their management of flower strips to maximise resources and reduce problematic weeds. Such advice should be made available for no extra cost for the farmers [81].

In the longer run, flower-strip networks may play an important role within the strategy of ecological intensification to combine high food production with the protection of a diverse flora and fauna. The applicability of this strategy and economical sustainability throughout Europe was recently demonstrated and discussed [25].

## Additional files

**Additional file 1.** Land-use in the study areas in 2015. Pattern varied from year to year but the type of crops and cover remained relatively stable over the years of the study.

**Additional file 2.** Chronology of seeding of the flower strips in the enhancement areas in Dettenheim and Rheinmünster. Spring sowing took place between April to the beginning of May, autumn sowing between September and the beginning of October. Annual seed mixtures are marked in light green, mixtures remaining over winter in middle dark green and perennial mixtures in dark green. If seed mixtures did not develop well, e.g. after the dry spring in 2013; even perennial mixtures were re-seeded earlier than planned. Sowing in autumn turned out very helpful to eliminate noxious weeds that germinate in spring and provided early flowering species for early flying pollinators.

**Additional file 3.** Seed mixtures used for the flower-strips between 2011 and 2015. For the chronology of use see Additional file 2.

**Additional file 4.** Original data on wild bee observations between 2010 and 2015. The observations are split up into female and male individual counts.

**Additional file 5.** Original data of butterfly observations in 2010 and 2015. 1 indicates the presence of the species.

## Abbreviations

CAP: common agricultural policies of the European Union
D: Delta or difference of the data from enhancement plots minus data from the control plots
