# Supplementary material for: Flower strip networks offer promising long term effects on pollinator species richness in intensively cultivated agricultural areas
Source: BMC Ecol. 2018 Dec 4;18:55. doi: 10.1186/s12898-018-0210-z (PMC6280486; doi:10.1186/s12898-018-0210-z)
Supplement: Supplementary file 1 — Additional file 1. Land-use in the study areas in 2015. Pattern varied from year to year but the type of crops and cover remained relatively stable over the years of the study. [file 12898_2018_210_MOESM1_ESM.docx]

Additional file 1:

|  | **Birkenhof / Rheinmünster** | | **Bolzhof / Dettenheim** | |
| --- | --- | --- | --- | --- |
|  | **Enhancement area** | **Control area** | **Enhancement area** | **Control area** |
| Agricultural fields | 95.6% | 96.4% | 95.7% | 93.4% |
| Grassland | 0.0% | 0.0% | 0.0% | 0.4% |
| Landscape elements | 3.8% | 2.5% | 3.9% | 5.6% |
| Roads | 0.5% | 1.1% | 0.4% | 0.6% |
| **Total area** | **50.7 ha** | **50.5 ha** | **50.7 ha** | **50.6 ha** |

| **Crop details** | **Birkenhof / Rheinmünster** | | **Bolzhof / Dettenheim** | |
| --- | --- | --- | --- | --- |
|  | **Enhancement area** | **Control area** | **Enhancement area** | **Control area** |
| Maize | 78.1% | 81.9% | 45.0% | 41.2% |
| Cereals | 8.4% | 16.5% | 34.3% | 51.1% |
| Flower strips | 10.3% | 1.6% | 10.2% | 0.0% |
| Others | 3.2% | 0.0% | 10.5% | 7.7% |
