# Supplementary material for: Flower strip networks offer promising long term effects on pollinator species richness in intensively cultivated agricultural areas
Source: BMC Ecol. 2018 Dec 4;18:55. doi: 10.1186/s12898-018-0210-z (PMC6280486; doi:10.1186/s12898-018-0210-z)
Supplement: Supplementary file 2 — Additional file 2. Chronology of seeding of the flower strips in the enhancement areas in Dettenheim and Rheinmünster. Spring sowing took place between April to the beginning of May, autumn sowing between September and the beginning of October. Annual seed mixtures are marked in light green, mixtures remaining over winter in middle dark green and perennial mixtures in dark green. If seed mixtures did not develop well, e.g. after the dry spring in 2013; even perennial mixtures were re-seeded earlier than planned. Sowing in autumn turned out very helpful to eliminate noxious weeds that germinate in spring and provided early flowering species for early flying pollinators. [file 12898_2018_210_MOESM2_ESM.pdf]

Chronology of seeding Dettenheim

<table>
<tr><th colspan="2" rowspan="2">Flower strip number</th><th rowspan="2">area in ha</th><th colspan="2">2011</th><th colspan="2">2012</th><th colspan="2">2013</th><th colspan="2">2014</th><th colspan="2">2015</th></tr>
<tr><th>spring</th><th>autumn</th><th>spring</th><th>autumn</th><th>spring</th><th>autumn</th><th>spring</th><th>autumn</th><th>spring</th><th>autumn</th></tr>
<tr><td></td><td></td><td></td><td>A
8kg/ha</td><td colspan="3">J
10kg/ha</td><td colspan="2">G
10kg/ha</td><td>H
10kg/ha</td><td colspan="3">O
10kg/ha</td></tr>
<tr><td>2</td><td></td><td>0.55</td><td colspan="2">B
10kg/ha</td><td colspan="2">A
8kg/ha</td><td colspan="2">E
7kg/ha</td><td>M</td><td colspan="3">O
10kg/ha</td></tr>
<tr><td>3</td><td></td><td>0.72</td><td colspan="2">A
8kg/ha</td><td colspan="2">D
12kg/ha</td><td colspan="2">L
20kg/ha</td><td colspan="4">L</td></tr>
<tr><td>4</td><td>west</td><td>0.59</td><td>C
30kg/ha</td><td>I
50kg/ha</td><td colspan="2">B
10kg/ha</td><td colspan="2">N
20kg/ha</td><td colspan="4">N
20kg/ha</td></tr>
<tr><td>4</td><td>east</td><td>0.69</td><td>C
30kg/ha</td><td>I
50kg/ha</td><td>B
10kg/ha</td><td colspan="3">J
10kg/ha</td><td colspan="4">H
10kg/ha</td></tr>
<tr><td>5</td><td>north</td><td>0.45</td><td colspan="2">D
12kg/ha</td><td>A
8kg/ha</td><td colspan="3">I
20kg/ha</td><td colspan="4">I
20kg/ha</td></tr>
<tr><td>5</td><td>south</td><td>0.48</td><td colspan="2">D
12kg/ha</td><td>A
8kg/ha</td><td colspan="3">I
20kg/ha</td><td colspan="2">I
20kg/ha</td><td colspan="2">J
10 kg/ha</td></tr>
<tr><td>6</td><td></td><td>0.33</td><td colspan="2">B
10kg/ha</td><td colspan="2">C
30kg/ha</td><td colspan="2">K
10kg/ha</td><td>J
10kg/ha</td><td colspan="3">O
10kg/ha</td></tr>
<tr><td>7</td><td></td><td>0.33</td><td>D
12kg/ha</td><td colspan="3">J
10kg/ha</td><td colspan="2">G
10kg/ha</td><td>G
10kg/ha</td><td colspan="3">O
10kg/ha</td></tr>
<tr><td>8</td><td></td><td>0.22</td><td colspan="2">D
12kg/ha</td><td>D
12kg/ha</td><td colspan="4">F
10kg/ha</td><td colspan="3">F
10kg/ha</td></tr>
<tr><td>9</td><td>north</td><td>0.23</td><td colspan="2">B
10kg/ha</td><td colspan="2">C
30kg/ha</td><td colspan="2">G
10kg/ha</td><td>G
10kg/ha</td><td colspan="3">O
10kg/ha</td></tr>
<tr><td>9</td><td>south</td><td>0.31</td><td colspan="2">B
10kg/ha</td><td colspan="2">C
30kg/ha</td><td colspan="2">G
10kg/ha</td><td colspan="4">G
10kg/ha</td></tr>
</table>

Chronology of seeding Rheinmünster

<table>
<tr><th rowspan="2">Flower strip number</th><th rowspan="2">area in ha</th><th colspan="2">2011</th><th colspan="2">2012</th><th colspan="2">2013</th><th colspan="2">2014</th><th colspan="2">2015</th></tr>
<tr><th>spring</th><th>autumn</th><th>spring</th><th>autumn</th><th>spring</th><th>Autumn</th><th>Spring</th><th>autumn</th><th>spring</th><th>autumn</th></tr>
<tr><td>1</td><td>0.31</td><td colspan="2">M</td><td colspan="2">C
30kg/ha</td><td>K
10kg/ha</td><td colspan="5">F
10kg/ha</td></tr>
<tr><td>2</td><td>0.55</td><td>D
12kg/ha</td><td colspan="2">I
50kg/ha</td><td colspan="2">I
50kg/ha</td><td colspan="2">H
10kg/ha</td><td colspan="3">O
10kg/ha</td></tr>
<tr><td>3</td><td>0.33</td><td colspan="2">D
12kg/ha</td><td colspan="2">D
12kg/ha</td><td>K
10kg/ha</td><td colspan="5">I
20kg/ha</td></tr>
<tr><td>4</td><td>0.21</td><td>M</td><td colspan="2">J
10kg/ha</td><td colspan="7">F
10kg/ha</td></tr>
<tr><td>5</td><td>0.69</td><td colspan="2">M</td><td colspan="2">C
30kg/ha</td><td>N
20kg/ha</td><td colspan="3">J
10kg/ha</td><td colspan="2">J
10kg/ha</td></tr>
<tr><td>6</td><td>0.89</td><td colspan="2">B
10kg/ha</td><td colspan="2">B
10kg/ha</td><td>E
7kg/ha</td><td colspan="2">N
20kg/ha</td><td colspan="3">O
10kg/ha</td></tr>
<tr><td>7</td><td>0.44</td><td>D
12kg/ha</td><td colspan="2">J
10kg/ha</td><td colspan="2">J
10kg/ha</td><td colspan="2">E
7kg/ha</td><td colspan="3">O
10kg/ha</td></tr>
<tr><td>8</td><td>0.61</td><td colspan="2">A
8kg/ha</td><td colspan="2">D
12kg/ha</td><td>L
20kg/ha</td><td colspan="2">L
20kg/ha</td><td colspan="3">O
10kg/ha</td></tr>
<tr><td>9</td><td>0.78</td><td colspan="2">C
30kg/ha</td><td colspan="2">A
8kg/ha</td><td>G
10kg/ha</td><td colspan="2">K
10kg/ha</td><td colspan="3">O
10kg/ha</td></tr>
<tr><td>10</td><td>0.21</td><td colspan="2">C
30kg/ha</td><td colspan="2">A
8kg/ha</td><td>G
10kg/ha</td><td colspan="2">G
20kg/ha</td><td colspan="3">O
10kg/ha</td></tr>
<tr><td>11</td><td>0.5</td><td colspan="8"></td><td colspan="2">P
15 kg/ha</td></tr>
</table>

Seed mixtures commercial and own mixtures: A "Tübinger Mischung"; B "MEKA 1"; C "Visselhöveder Insektenparadies"; D "Kultur-Natur-Blüht-auf"; E "Göttinger Mischung"; F "Blühende landschaft Süd"; G "IFAB 1"; H "IFAB 2"; I "Leguminosenmischung"; J "Brassicaceae Mischung"; K "Biogas"; L "Wildbienen A"; M mixed seeds from other mixtures of variable quantities; N "Wildbienen B"; O "Oberrhein überjährig"; P " FAKTM2"
