# Supplementary material for: Flower strip networks offer promising long term effects on pollinator species richness in intensively cultivated agricultural areas
Source: BMC Ecol. 2018 Dec 4;18:55. doi: 10.1186/s12898-018-0210-z (PMC6280486; doi:10.1186/s12898-018-0210-z)
Supplement: Supplementary file 3 — Additional file 3. Seed mixtures used for the flower-strips between 2011 and 2015. For the chronology of use see Additional file 2. [file 12898_2018_210_MOESM3_ESM.pdf]

Seed mixtures applied during the enhancement period between 2011 and 2015

Compare to supplement 2 for application periods

A: „Tübinger Mischung“

Plant	Weight %
Anethum graveolens	2
Borago officinalis	1
Calendula officinalis	5
Centaurea cyanus	3
Coriandrum sativum	6
Fagopyrum esculentum	25
Malva verticillata	3
Nigella sativa	5
Phacelia tanacetifolia	40
Raphanus sativus	3
Sinapis alba	7

B: „Meka I“

Plant	Weight %
Borago officinalis	5
Calendula officinalis	2
Centaurea cyanus	2
Fagopyrum esculentum	40
Helianthus annuus	8
Linum usitatissimum	9
Malva sylvestris ssp. mauretania	1
Papaver rhoeas	1
Phacelia tanacetifolia	12
Trifolium incarnatum	10
Trifolium resupinatum	10

C: „Visselhöveder Insektenparadies“

Plant	Weight %
Anethum graveolens	3
Borago officinalis	2
Coriandrum sativum	2
Fagopyrum esculentum	35
Helianthus annuus	9
Linum usitatissimum	14
Malva verticillata	2
Ornithopus sativus	3
Phacelia tanacetifolia	12
Raphanus sativus	4
Sinapis alba	7
Trifolium alexandrinum	3
Trifolium resupinatum	3
Vicia sativa	3

D: „Kultur-Natur blüht auf“

Plant	Weight %
Anethum graveolens	3
Borago officinalis	2
Fagopyrum esculentum	24
Phacelia tanacetifolia	7
Coriandrum sativum	3
Trifolium incarnatum	4
Foeniculum vulgare	5
Linum usitatissimum	12
Helianthus annuus	13
Anethum graveolens	3
Calendula officinalis	5
Centaurea cyanus	5
Lepidium sativum	4
Onobrychis viciifolia	5
Papaver rhoeas	1
Vicia sativa	5

E: „Göttinger Mischung“

Plant	Weight %
Avena sativa	5
Borago officinalis	5
Brassica oleracea	0.5
Fagopyrum esculentum	15
Foeniculum vulgare	5
Helianthus annuus	15
Lepidium sativum	0.5
Linum usitatissimum	15
Malva sylvestris ssp. mauritiana	5
Medicago sativa	7
Melilotus officinalis	2
Onobrychis viciifolia	5
Phacelia tanacetifolia	7
Raphanus sativus	7
Secale multicaule	5
Sinapis alba	1

F: „Blühende Landschaft Süd“

Plant	Weight %
Achillea millefolium	*1.0*
Allium fistulosum	*2.0*
Anthemis tinctoria	*1.5*
Borago officinalis	*2.0*
Calendula officinalis	*7.0*
Campanula rapunculoides	*0.2*
Centaurea cyanus	*5.8*

Centaurea jacea	*1.5*
Cichorium intybus	*2.0*
Daucus carota	*2.0*
Echium vulgare	*3.0*
Fagopyrum esculentum	*9.0*
Helianthus annuus	*13.0*
Hypericum perforatum	*0.5*
Isatis tinctoria	*0.5*
Knautia arvensis	*0.5*
Leucanthemum vulgare	*3.0*
Linum usitatissimum	*9.0*
Lotus corniculatus	*0.8*
Malva moschata	*0.5*
Medicago lupulina	*2.0*
Medicago sativa	*3.0*
Melilotus alba	*0.3*
Melilotus officinalis	*0.3*
Onobrychis viciifolia	*3.5*
Origanum vulgare	*0.2*
Papaver rhoeas	*2.0*
Pastinaca sativa	*1.5*
Phacelia tanacetifolia	*5.0*
Plantago lanceolata	*2.0*
Reseda lutea	*0.3*
Salvia pratensis	*1.5*
Sanguisorba minor	*2.0*
Silene dioica	*1.0*
Silene latifolia ssp. alba	*1.0*
Sinapis alba	*2.0*
Sinapis arvensis	*1.5*
Solidago virgaurea	*0.3*
Tanacetum vulgare	*0.1*
Trifolium incarnatum	*2.0*

Verbascum densiflorum	*0.5*
Vicia sativa	*3.2*

G: „IFAB I“

**Plant**	**Weight %**
Achillea millefolium	0.2
Agrostemma githago	3
Anthyllis vulneraria	2
Borago officinalis	5
Brassica napus	4
Carum Carvi	2
Centaurea cyanus	1.5
Centaurea jacea	0.1
Coriandrum sativum	7
Daucus carota	1
Echium vulgare	0.2
Fagopyrum esculentum	12
Foeniculum vulgare	5
Lepidium sativum	2
Leucanthemum vulgare	0.5
Lotus corniculatus	1
Medicago sativa	3
Melilotus alba	0.25
Melilotus officinalis	1
Onobrychis viciifolia	3
Origanum vulgare	0.1
Papaver rhoeas	0.1
Phacelia tanacetifolia	1
Plantago lanceolata	1
Secale multicaule	25
Sinapis alba	2
Trifolium pratense	2

Verbascum densiflorum	0.05
Vicia sativa	5
Vicia villosa	10

H: „IFAB II“

**Plant**	**Weight %**
Achillea millefolium	0.5
Agrostemma githago	10
Anethum graveolens	1
Anthemis tinctoria	1
Borago officinalis	4
Brassica napus	1.5
Calendula officinalis	7
Centaurea cyanus	1.5
Cichorium intybus	3
Coriandrum sativum	7
Daucus carota	1.5
Dipsacus fullonum	2
Eruca sativa (rucola coltivata)	1
Fagopyrum esculentum	9
Foeniculum vulgare	3
Linaria vulgaris	0.4
Linum usitatissimum	6
Lotus corniculatus	3
Malva sylvestris ssp. mauritiana	2
Medicago lupulina	3
Medicago sativa	1.5
Melilotus officinalis	2.5
Onobrychis viciifolia	10
Papaver rhoeas	0.2
Phacelia tanacetifolia	0.5
Raphanus sativus	0.5

Securigera varia	1.5
Silene alba	2
Silybum marianum	8
Sinapis alba	1
Sinapis arvensis	1
Tanacetum vulgare	0.4
Trifolium incarnatum	2.5
Trifolium pratense	0.5
Trifolium repens	0.5

I: „Leguminosenmischung“

Plant	Weight %
Medicago lupulina	10
Medicago sativa	20
Melilotus officinalis	5
Onobrychis viciifolia	20
Papaver rhoeas	3
Trifolium incarnatum	7
Trifolium pratense	15
Trifolium repens	20

J: „Brassicaceae-Mischung“

Plant	Weight %
Achillea millefolium	5
Brassica napus	10
Brassica rapa var. rapa	10
Carum carvi	10
Coriandrum sativum	15
Foeniculum vulgare	5
Malva sylvestris ssp. mauritiana	5
Onobrychis viciifolia	6
Papaver rhoeas	1
Raphanus sativus	3
Secale multicaule	20
Sinapis alba	2
Sinapis arvensis	3
Vicia villosa	5
Achillea millefolium	5
Brassica napus	10
Brassica rapa var. rapa	10

K: „Biogas“

Plant	Weight %
Fagopyron esculentum	4
Guizotia abyssinica	2
Malva verticiliata	6
Helianthus annus	18
Althaea officinalis	5,5
Anthemis tinctoria	0,1
Artemisia vulgaris	0,5
Centaurea nigra	18
Cichorium intybus	1
Daucus carota	0,1
Dipsacus sylvestris	0,5
Echium vulgare	0,5
Foeniculum vulgare	1
Inula helenium	4
Malva alcea	0,5
Malva mauritanica sylva	3
Malva sylvestris	8
Medicago sativa	2
Melilotus albus	3,5
Melilotus officinalis	3,5
Onobrychis viciifolia	7
Reseda luteola	0,3
Silene alba	0,1
Silene dioica	0,2
Tanacetum vulgare	3,5
Verbascum thapsus	0,2

L: „Wildbienen A“

Plant	Weight %
Borago officinalis	2
Brassica napus	1.5
Brassica oleracea	1.5
Calendula officinalis	5
Camelina sativa	0.5
Campanula rapunculoides	0.25
Centaurea cyanus	10
Coriandrum sativum	5.5
Hypochoeris radicata	1
Knautia arvensis	1
Leucanthemum ircutianum	1
Linum usitatissimum	15
Lotus corniculatus	2
Malva sylvestris	4
Medicago sativa	2.5
Onobrychis viciifolia	13
Papaver rhoeas	2.5
Picris hieracoides	0.5
Pisum sativum	10
Raphanus sativus oleiformis	1.5
Reseda lutea	0.5
Sinapis alba	1
Sinapis arvense	2
Stachys palustris	1
Trifolium incarnatum	2.5

Trifolium pratense (wild form)	2.5
Vicia faba	10.25

N: „Wildbienen B“

Plant	Weight %
Borago officinalis	4
Calendula officinalis	6.25
Campanula rapunculoides	0.25
Centaurea cyanus	5
Coriandrum sativum	5
Hypochoeris radicata	1
Knautia arvensis	1
Leucanthemum ircutianum	2.5
Linum usitatissimum	25
Lotus corniculatus	2.5
Medicago sativa	4
Onobrychis viciifolia	12.5
Papaver rhoeas	0.75
Pisum sativum	14.75
Picris hieracoides	1
Reseda lutea	1
Stachys palustris	1
Trifolium incarnatum	2.5
Trifolium pratense (wild form)	2.5
Vicia faba	7.5

O: „Oberrhein überjährig 2014-2015“

Plant	Weight %
Achillea millefolium	0.5
Agrostemma githago	6
Althaea officinalis	1
Anethum graveolens	3
Anthemis tinctoria	1
Borago officinalis	2
Brassica napus	5
Brassica oleracea	1
Calendula officinalis	7
Carum Carvi	3
Centaurea cyanus	3
Cichorium intybus	1
Consolida regalis	0.5
Coriandrum sativum	10
Daucus carota	1
Fagopyrum esculentum	10
Foeniculum vulgare	4
Helianthus annuus	7
Leonurus cardiaca	0.5
Lepidium sativum	2
Leucanthemum vulgare	1
Lotus corniculatus	4
Malva sylvestris	1
Medicago sativa	5
Melilotus officinalis	1
Papaver rhoeas	0.5
Phacelia tanacetifolia	2
Raphanus sativus	2
Reseda luteola	0.5
Sinapis alba	4
Sinapis arvensis	0.5
Trifolium incarnatum	4
Trifolium pratense	1
Vicia sativa	5

P: „FAKT M2“

Plant	Weight %
Phacelia tanacetifolia	12
Fagopyrum esculentum	0
Guizotia abyssinica	7,5
Sinapsis alba	0
Calendula officinalis	6
Raphanus sativus	0
Borago officinalis	3
Linum usitatissimum	10
Trifolium resupinatum	5
Helianthus annuus	17
Trifolium incarnatum	10
Centaurea cyanus	6
Papaver rhoeas	0,5
Coriandrum sativum	5
Anethum graveolens	2
Vicia sativa	6
Onobrychis viciifolia	5
Foeniculum vulgare	5
